# Patterns of Spatial Variation of Assemblages Associated with Intertidal Rocky Shores: A Global Perspective

**DOI:** 10.1371/journal.pone.0014354

**Published:** 2010-12-16

**Authors:** Juan José Cruz-Motta, Patricia Miloslavich, Gabriela Palomo, Katrin Iken, Brenda Konar, Gerhard Pohle, Tom Trott, Lisandro Benedetti-Cecchi, César Herrera, Alejandra Hernández, Adriana Sardi, Andrea Bueno, Julio Castillo, Eduardo Klein, Edlin Guerra-Castro, Judith Gobin, Diana Isabel Gómez, Rafael Riosmena-Rodríguez, Angela Mead, Gregorio Bigatti, Ann Knowlton, Yoshihisa Shirayama

**Affiliations:** 1 Departamento de Estudios Ambientales, Universidad Simón Bolívar, Caracas, Miranda, Venezuela; 2 Laboratorio de Ecosistemas Costeros, Museo Argentino de Ciencias Naturales “Bernardino Rivadavia”, Buenos Aires, Argentina; 3 School of Fisheries and Ocean Sciences, University of Alaska Fairbanks, Fairbanks, Alaska, United States of America; 4 The Huntsman Marine Science Centre, St. Andrews, New Brunswick, Canada; 5 Suffolk University, Boston, Massachusetts, United States of America; 6 Dipartimento di Biologia, University of Pisa, Pisa, Tuscany, Italy; 7 Centro de Biodiversidad Marina, Universidad Simón Bolívar, Caracas, Miranda, Venezuela; 8 Centro de Ecología, Instituto Venezolano de Investigaciones Científicas, Caracas, Miranda, Venezuela; 9 Department of Life Sciences, The University of The West Indies, St. Augustine, Trinidad and Tobago; 10 Instituto de Investigaciones Marinas y Costeras “Jose Benito Vives De Andreis”, Santa Marta, Magdalena, Colombia; 11 Programa de Investigación en Botánica Marina, Universidad Autónoma de Baja California Sur, La Paz, México; 12 University of Cape Town, Cape Town, Western Cape, South Africa; 13 Centro de Estudios Nacionales Patagónicos, Puerto Madryn, Chubut, Argentina; 14 Seto Marine Biological Laboratory, Field Science Education and Research Center, Kyoto University, Shirahama, Wakayama, Japan; National Institute of Water & Atmospheric Research (NIWA), New Zealand

## Abstract

Assemblages associated with intertidal rocky shores were examined for large scale distribution patterns with specific emphasis on identifying latitudinal trends of species richness and taxonomic distinctiveness. Seventy-two sites distributed around the globe were evaluated following the standardized sampling protocol of the Census of Marine Life NaGISA project (www.nagisa.coml.org). There were no clear patterns of standardized estimators of species richness along latitudinal gradients or among Large Marine Ecosystems (LMEs); however, a strong latitudinal gradient in taxonomic composition (i.e., proportion of different taxonomic groups in a given sample) was observed. Environmental variables related to natural influences were strongly related to the distribution patterns of the assemblages on the LME scale, particularly photoperiod, sea surface temperature (SST) and rainfall. In contrast, no environmental variables directly associated with human influences (with the exception of the inorganic pollution index) were related to assemblage patterns among LMEs. Correlations of the natural assemblages with either latitudinal gradients or environmental variables were equally strong suggesting that neither neutral models nor models based solely on environmental variables sufficiently explain spatial variation of these assemblages at a global scale. Despite the data shortcomings in this study (e.g., unbalanced sample distribution), we show the importance of generating biological global databases for the use in large-scale diversity comparisons of rocky intertidal assemblages to stimulate continued sampling and analyses.

## Introduction

The study of biological diversity or biodiversity has gained strong scientific interest in recent decades (13,029 and 31,691 references, respectively, in Web of Science in the last decade), due to the consequences that diversity loss might have on humanity [1]. Compelling evidence signals that our climate is changing [2] and is driving important shifts in the composition and structure of a diverse array of natural assemblages: terrestrial [3], marine [4]–[6], aquatic [7] and pathogens [8]. Given the close relationship between biodiversity and the ecosystem function [9]–[11], any diversity loss will be negatively reflected in the number and quality of services that a particular system might provide [12]–[14]. Consequently, it is of paramount importance to be able to detect these types of changes in natural ecosystems.

To detect changes in natural communities, and unequivocally relate them to anthropogenic impacts or climate disruptions, proper biological baseline data are of utmost importance. Very few long-term/large-scale data sets are currently available (but see [15] as an example), and comparison of other existing data is often hampered by differing methodologies. Consequently, standardized global monitoring programs need to be implemented to assess changes in biodiversity and relate those changes to possible causes. Out of this need, the Census of Marine Life (CoML) NaGISA project (Natural Geography in Shore Areas, [16]) was initiated in 2002 with the main objective of inventorying coastal biodiversity on a global scale. NaGISA's strength is the use of a standardized sampling protocol by a closely interconnected global network of scientists that can allow for comparisons at different spatial scales across the globe [17]. The NaGISA project focuses on assemblages associated with rocky shores and on those associated with soft-sediment seagrass beds. The present study focused on intertidal assemblages associated with rocky shores.

Intertidal rocky shores assemblages are appropriate to study changes driven by global-scale anthropogenic impacts and climate change effects due to their ecological characteristics and accessibility [18], [19]. Nevertheless, few studies have examined anthropogenic impacts on intertidal rocky shore assemblages at broad scales, (e.g., [20]–[23]). Most were limited to regional scales including the US west coast (e.g., [23]), United Kingdom [15], Portugal [24], Japan [21], and Mediterranean Sea [16]. Given that strong differences exist among regions in terms of anthropogenic and climate change impacts, e.g., different warming rates [25], [26] and human influence [27], a global-scale approach is warranted. Consequently, the main objective of this study was to quantitatively describe the distribution and diversity patterns of intertidal rocky shores assemblages at globally distributed sampling locations as a baseline that might be used in the future to detect changes, and to relate these changes to possible drivers of change.

Current paradigms of latitudinal diversity gradients postulate an increasing number of species increases from the pole to the equator [28]. Although recent meta-analysis suggests that this trend can be viewed as a generalized pattern in marine taxa [29], [30], there are exceptions for particular taxa and ecosystems, e.g., for macroalgae [31]–[33] and for soft-sediment shelf communities [34]. Consequently, the first objective of this study was to asses latitudinal trends in species richness of assemblages associated with intertidal rocky shores. Description of such large-scale trends alone does not, however, elucidate the potential mechanisms that might be responsible for the described patterns. Three different models may explain the spatial distribution patterns of natural assemblages at large scales. One model postulates that biological interactions at small spatial scales (e.g., meters) influence communities, and as such, under these so-called null models it is hypothesized that species composition is uniform over large areas (i.e., [35]). The second model postulates that larval dispersal and supply are the driving mechanisms; therefore, neutral models predict that species composition fluctuates in a random, autocorrelated way [36]–[38]. The third model postulates that abiotic factors structure communities, and these environmental models hypothesize that species distributions are related to environmental conditions [39], [40] and/or sources of human impact [41], [42]. Therefore, in an attempt to elucidate the relevance of these alternative models, the second objective of this study was to relate rocky intertidal assemblage structure with several environmental variables linked to anthropogenic or natural influences.

## Materials and Methods

This study was carried out as part of the research conducted by the Laboratorio de Ecología Experimental, approved by and under the guidelines of the Departamento de Estudios Ambientales, Universidad Simón Bolívar, Caracas, Venezuela.

### Study sites

**Figure 1:**
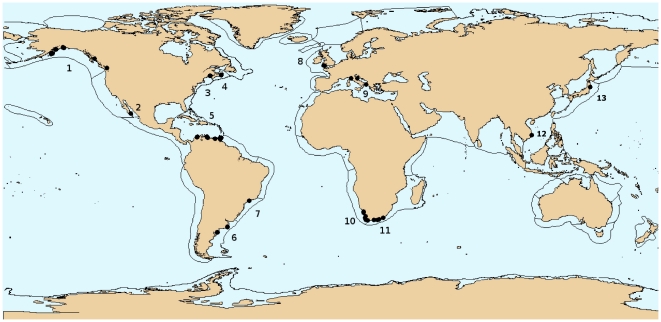
Global distribution of sampling sites within Large Marine Ecosystems (LMEs). 1 =  Gulf of Alaska, 2 =  Gulf of California, 3 =  Northeast U.S. Continental Shelf, 4 =  Scotian Shelf, 5 =  Caribbean Sea, 6 =  Patagonian Shelf, 7 =  South Brazilian Shelf, 8 =  Celtic-Biscay Shelf, 9 =  Mediterranean Sea, 10 =  Benguela Current, 11 =  Aghulas Current, 12 =  South China Sea, 13 =  Kuroshio Current.

Surveys were done at 72 rocky intertidal sites (Fig. 1) distributed across the globe and were grouped into 13 Large Marine Ecosystems (LMEs) as defined by [43] (Table 1). LMEs are large areas of ocean space (≈ 200,000 km^2^ or greater) in coastal waters where primary productivity is generally higher than in the open ocean. LME boundaries are based on bathymetry, hydrography, productivity regime and trophic relationships [43]. The LME concept was selected because it is a tool that has enabled ecosystem-based management in at least 16 international projects across the world [43]. Sites were sampled between June 2004 and January 2009 and included a variety of site-specific characteristics (Table 1). Given that different regions were not sampled at the same time, caution is warranted when comparing different sites and LMEs because estimates of temporal variation are lacking.

**Table 1:** Description of Large Marine Ecosystems (LME) indicating number of sites sampled per LME's and general characteristics.

LMEs	Abb.	Sites	Replicates per site	Ocean	Countries	Bottom type
Gulf of Alaska	GoA	11	5	Pacific	USA, Canada	Bedrock, Sandstone and Boulders
Agulhas Current	AgC	7	10	Indian	South Africa	Boulders and Sandstone
Celtic-Biscay Shelf	CBS	2	5	Atlantic	England	Bedrock
Northeast U.S Continental Shelf	NCS	2	5	Atlantic	USA, Canada	Cobbles and Bedrock
Caribbean Sea	CbS	29	10	Atlantic	Colombia, Venezuela, Trinidad & Tobago	Bedrock
Benguela Current	BgC	7	10	Atlantic	South Africa	Boulders, Sandstone and Rocky reef
Mediterranean Sea	MdS	3	5	Mediterranean Sea	Italy	Bedrock and Sandstone
Scotian Shelf	StS	1	5	Atlantic	Canada	Cobbles
South China Sea	SCS	1	3	Pacific	Vietnam	Bedrock
Patagonian Shelf	PaS	5	10	Atlantic	Argentina	Bedrock
Kuroshio Current	KuC	1	5	Pacific	Japan	Bedrock
South Brazil Shelf	SBS	1	5	Atlantic	Brazil	Bedrock
Gulf of California	GoC	2	5	Pacific	Mexico	Loose boulders

Abb  =  Abbreviation code for LMEs, SST  =  Sea Surface Temperature.

### Biological sampling

Data were collected following the standardized NaGISA protocol [44]. This study only used data from the mid-low intertidal zone to reduce the effects of the wide variation in tidal amplitude among globally distributed sites, ranging from about 30 cm in the Caribbean to 7+ m in the Bay of Fundy and the Gulf of Alaska. At each site, 5 to 10 randomly placed 1 m^2^ quadrats were sampled with nondestructive methods along 30 m transects positioned parallel to the waterline in the mid and/or low intertidal zone. Abundance of macroalgae and colonial fauna were estimated by percent cover and individuals (>2 cm) were counted. Most identification were made in the field on living organisms, although occasional problematic specimens were collected for reference and sent to specialists for identification. All organisms were identified to the lowest taxon possible, which in most cases was species. Percentage cover and counts were restricted to visible organisms living on the surface and not beneath rocks.

### Environmental data

Fourteen environmental variables were examined to determine the most important drivers for describing trends in species numbers and composition of assemblages associated with intertidal rocky shores. Variables were estimated for sampling site within each LME using different sources and were grouped into variables related to either “natural” or “anthropogenic” influences (Table 2). This classification separates those variables that are **directly** related with anthropogenic causes vs. those that are not **directly** related to them.

**Table 2:** List of environmental variables used in analysis.

Variable	Short	Description	Reference
**Natural**			
Sea-surface temperature	SST	Average of monthly values of the MODIS Aqua mission from July 2002 to December 2009	
Chlorophyll-*a*	CHA	Average of monthly values of the MODIS Aqua mission from July 2002 to December 2009	
Chlorophyll-*a* anomalies	CHAa	Numbers of events that surpassed 2 standard deviations of the average chlorophyll-a for a given year	
Rainfall	RAI	Average of monthly accumulated rainfall from January 1979 through September 2009 obtained using the TOVAS web-based application	
Rainfall anomalies	RAIa	Numbers of events that surpassed 2 standard deviations of the average rainfall for a given year	
Photoperiod	PHO	Common astronomical formulae were used to compute the difference between the sunrise and sunset time	[92]
**Anthropogenic**			
Inorganic pollution	INP	Urban runoff estimated from land-use categories, US Geologic Survey (http://edcsns17.cr.usgs.gov/glcc/)	[45]
Organic pollution	ORP	FAO national pesticides statistics (1992–2001), (http://faostat.fao.org)	[45]
Nutrient contamination	NUTC	FAO national fertilizers statistics (1993–2002), (http://faostat.fao.org)	[45]
Acidification	AC	Aragonite saturation state 1870–2000/2009, 1 degree lat/long resolution	[45]
Invasive species incidence	INV	Cargo traffic 1999–2003	[45]
Population pressure	HUM	Estimated as the sum of total population adjacent to the ocean within a 25 km radius. LandScan 30 arc-second population data of 2005 were used.	[45]
Shipping activity	SH	Commercial ship traffic 2004–2005	[45]
Ocean-based pollution	OBP	Modelled as a combination of commercial shipping traffic data and port data	[45]

Because of the coarse spatial resolution of the environmental data and the land-mask imposed to the models from which data were derived from, most variables were not predicted precisely for the shore sampling sites. When a site was no farther than 50 km from the model, a spline interpolation was used to the raster data to compute its value at the coordinate of the sampling site. Furthermore, LMEs were used as the scale of comparison in this study because of the potential inaccuracy of satellite-derived data from optical sea-surface properties (e.g., chlorophyll-a, primary productivity) on small spatial scales [45]. Environmental variables related to direct anthropogenic influences were collected at a 1 km resolution; however, the nearshore environment is highly variable and can be influenced by point sources. Combining site data by LMEs allowed to concentrate on large-scale variability, which has higher than the one found at smaller-scale, [Table S1, [46]].

### Data analyses

Since different sampling efforts were used in different LMEs, the number of taxa at each LME was interpolated using saturation curves (i.e., UGE method [47] for 999 permutations) for a standard sampling size of 20 replicates (1 m^2^ quadrats) per LME [48]. The number of taxa at each site was estimated using the same method of saturation curve, but in this case for a standard sampling size of 5 replicates (1 m^2^ quadrats). These estimates predict how many species would have been found in each LME or site if 20 (LME) or 5 (site) quadrats were sampled. In LMEs or sites where less than 20 or 5, respectively, quadrats were sampled (e.g., Vietnam and Japan, Table 1) these estimates were not calculated. Pearson correlation analyses were done between the estimators of species richness per site and latitude in order to detect possible patterns of distribution across latitudinal gradients.

Biological data from each site were transformed to presence-absence data and a similarity matrix was constructed based on the taxonomic dissimilarity coefficient *Theta* defined by Clarke and Warwick [49] and Clarke et al. [50]. This coefficient is particularly suitable to compare samples across large geographical scales that do not share many species. *Theta* takes into consideration the taxonomic relationship of species found in each sample, and consequently, two samples with no species in common, can have a dissimilarity value <100 [50]. Based on this dissimilarity matrix, the distances among centroids of sampling sites [51] were visualized using Canonical Analysis of Principal Coordinates (CAP) ordinations [52] and considering LME groups as the predictor variable. Families contributing the most to these differences were detected using SIMPER analyses [53], [54]. Similarity matrices on the species and family levels were correlated at ρ = 0.78, indicating that the family level preserved taxonomic dissimilarity patterns.

Environmental variables were normalized to a common scale. Geographic coordinates were included in this matrix and considered in further analyses in order to detect possible effects of distances among sampling sites. Redundant variables were identified using multiple correlation analysis (i.e., draftsman plots) after square-root transformation of skewed variables and excluded from the analysis. It is important to note that whenever latitude or longitude were used, these variables conserved their sign. To select the combination of variables that best matched the biological distribution patterns, a similarity matrix of environmental variables based on Euclidean distances was linked to the taxonomic dissimilarities patterns (*Theta* matrix) among LMEs using the BEST [55] routine. All procedures described here were done using the PRIMER-e [54] and PERMANOVA add-on [51] software.

## Results

A total of 801 taxa were identified from 1499 sample quadrats. The number of observed and standardized taxon richness varied among LMEs (Table 3). Based on standardized measures of richness (UGE), the highest values were found in the Caribbean Sea followed by the Agulhas and Benguela Current LMEs (Table 3). Most LMEs were dominated by algae. Exceptions were the Patagonian Shelf, which was dominated by mussel beds of *Brachidontes rodriguezii* and *Perumytilus purpuratus*, the South Brazil Shelf, which was dominated by the barnacle *Chthamalus bisinuatus*, and the site located in the Kuroshio Current, which was dominated by the sponge *Halichondria japonica* (Table 3). Encrusting coralline red algae dominated the Caribbean Sea, Agulhas Current, Benguela Current and Mediterranean Sea LMEs. The remaining LMEs were dominated by fucoid algae (Table 3). In terms of grazers, all sites in all LMEs were dominated by gastropods (mainly limpets), with the exception of the Caribbean Sea, where the main grazer was the sea urchin *Echinometra lucunter* (Table 3).

**Table 3:** General biological information for each LME.

LMEs	n	S	UGE (n = 20)	Dominant group	Grazers	Other important species
Gulf of Alaska	110	106	45	Brown and red algae (Phaeophyceae)	Littorinidae, limpets and chitons (Lottiidae and Littorina)	*Evasterias troscheli* (sea star) *Katharina tunicata* (chiton)
Agulhas Current	70	110	86	Red algae *(Spongites yendoi)*	Littorinidae and limpets *(Afrolittorina knysnaensis)*	*Gunnarea capensis* (polychaete) *Chthamalus dentatus* (barnacle) *Tetraclita serrata* (barnacle)
Celtic-Biscay Shelf	20	45	45	Brown and red algae *(Fucus spp)*	Littorinidae, limpets and snails *(Gibbula umbilicalis)*	*Patella vulgata* (limpet), *Osilinus lineatus* (snail) *Littorina spp* (snail)
Northeast U.S Continental Shelf	20	47	47	Brown algae *(Ascophyllum nodosum)*	Littorinidae and limpets *(Littorina littorea)*	*Balanus balanoides* (barnacle) *Tectura testudinalis* (limpet) *Mytilus edulis* (bivalve)
Caribbean Sea	154	261	120	Brown, red and green algae (encrusting coralline algae)	Littorinidae, sea urchins, limpets, snails and chitons *(Echinometra lucunter)*	*Acmea antillarum* (limpet) *Fissurella spp* (limpet)
Benguela Current	70	97	75	Brown and red algae *(Spongites yendoi)*	Littorinidae, limpets, snails and chitons *(Scutellastragranularis)*	*Mytilus galloprovincialis* (bivalve) *Gunnarea capensis* (polychaete) *Dodecaceria pulchra* (polychaete)
Mediterranean Sea	40	65	57	Brown and red algae (Corrallinaceae)	Littorinidae, Sea urchins, Limpets and Snails *(Patella spp)*	*Phorcus mutabilis* (snail) *Osilinus turbinatus* (snail)
Scotian Shelf*	10	7	n/d	Brown algae *(Fucus spp)*		
South Chine Sea*		7	n/d	Barnacle	Limpets (Patellogastropoda)	Saccostrea (bivalve)
Patagonian Shelf	59	35	30	Mussels *(Brachidontes rodriguezii)*	Limpets *(Siphonaria lessoni)*	*Balanus glandula* (barnacle) *Mytilus sp.* (bivalve) Actiniidae (sea anemone)
Kuroshio Current*	5	4	n/d	Sponges *(Halichondria japonica)*	Limpets and chitons *(Lottia dorsuosa)*	Patellogastropoda
South Brazil Shelf*	5	34	n/d	Barnacles *(Chthamalus bisinuatus)*		
Gulf of California	20	8	8	Cyanophyceae	Snails *(Nerita funiculata)*	*Chthamalus sp*.(barnacle) *Pilsbryspira nymphia* (snail)

Includes total number of quadrats (n), total number of observed taxa (S), estimators of number of taxa for a standard sampling size of 20 quadrats based on saturation curves (UGE method) and most common species or taxa per LME. Asterisks denote LMEs with fewer than 20 quadrats (n<20), for which no UGE was calculated (n/d)

**Figure 2:**
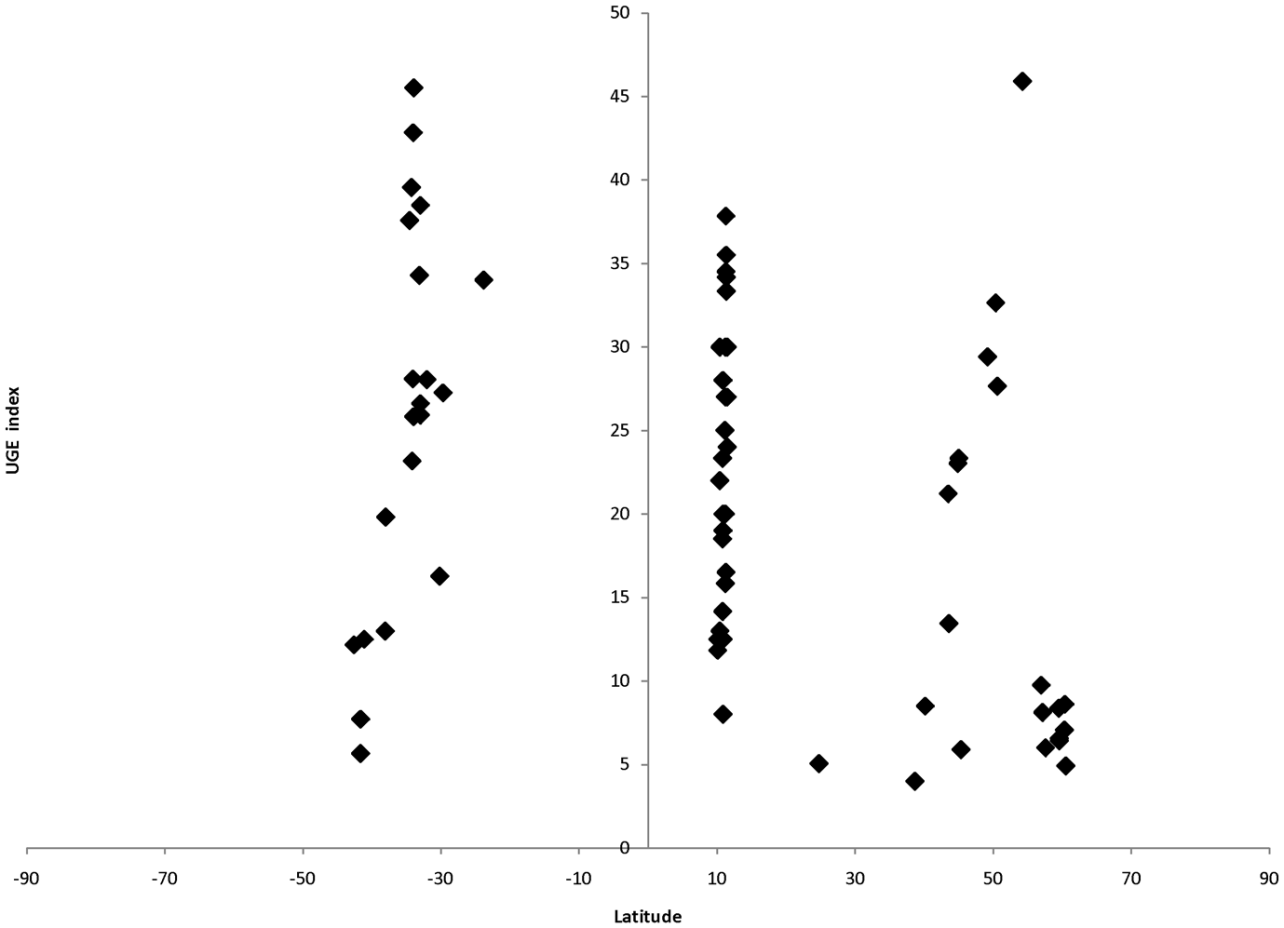
Latitudinal variations for standardized richness estimates per site (n = 5).

No latitudinal patterns were found using UGE-standardized richness estimates as indicated by a low Pearson's correlation index (ρ = −0.12; Fig. 2). Variation in standardized richness among sampling sites within the same latitudinal range was similar to that observed across the latitudinal gradient. For example, in the Caribbean Seas (≈10° north) and the Gulf of Alaska (≈60° north), sites with low and high values of standardized richness estimates occurred.

**Figure 3:**
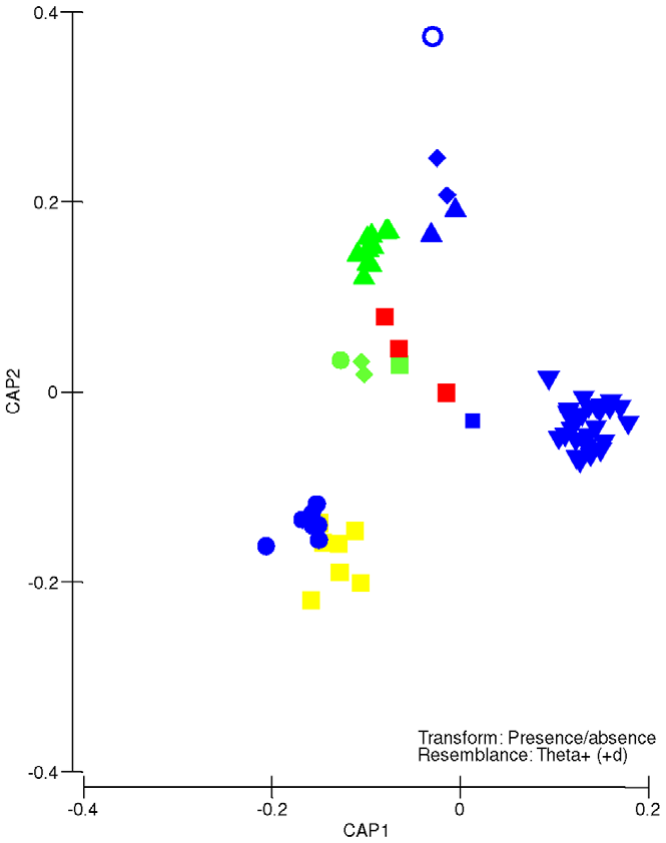
CAP on biological data. Canonical analysis of principal coordinates (CAP) plots generated from taxonomic dissimilarity coefficients (*theta*) of the biological data matrix, using LMEs as predictor factor. Green triangle  =  Gulf of Alaska, Yellow square  =  Agulhas Current, Red square  =  Mediterranean Sea, Blue triangle  =  Celtic-Biscay Shelf, Green diamond  =  Gulf of California, Blue diamond  =  Northeast US Continental Shelf, Inverted blue triangle  =  Caribbean Sea, Blue circle =  Benguela Current, Green square  =  South China Sea, Green circle  =  Kuroshio Current, Blue square  =  Patagonian Shelf, Empty blue circle  =  Scotian Shelf.

A constrained ordination (CAP) of sampling sites using LMEs as predictor factor effectively showed that sites based on assemblage information were strongly grouped by LME (Fig. 3). Sites within the Caribbean Sea LME showed the most conspicuous separation along the first axis, indicating very different taxonomic composition of species assemblages. In addition, sites were distributed mainly according to CAP2 (δ_2_ = 97.5%). This distribution along the second axis followed a close association with their relative latitudinal position. South African LME sites were plotted at the bottom of the ordination whereas those of the Scotian shelf LME were located at the top of the ordination (Fig. 3). In between, and from north to south, sites in the Gulf of Maine Northeast US Shelf and Celtic Shelf LMEs grouped together, and the Gulf of Alaska sites formed a tight cluster. Sites in the Mediterranean were ordered together with sites that were longitudinally very distant (i.e., sites of the Kuroshi Current LME), but located at relatively similar latitudes, around 38° to 41° north (Fig. 3). These results clearly show that the taxonomic composition of assemblages associated with intertidal rocky shores gradually changed in relation to latitude, which contrasts the lack of a relationship between standardized estimators of richness and latitude.

The latitudinal trend in taxonomic composition was largely due to the presence of prominent taxonomic groups in the LMEs as indicated by SIMPER analysis (Table 4). The Gulf of Alaska sites differed from others LMEs by the presence of various families of Phaeophyta, Rhodophyta and Chlorophyta (Table 4). Encrusting forms of algal families (i.e., mainly Corallinaceae and Rhodomelaceae) were more important in the South-African (Agulhas and Benguela Current), Caribbean Sea and Mediterranean Sea LMEs than elsewhere. These LMEs with abundant encrusting algae, were dominated by different grazers, i.e., sea urchins in the Caribbean, patellid gastropods in the Mediterranean Sea, and both patellid and siphonarian gastropods in the South-African LMEs. Fucoids were more important on the Northeast US Shelf, Celtic-Biscay Shelf and Scotian Shelf compared to other LMEs. Barnacles distinguished the Gulf of California and South China Sea LMEs from the rest. (Table 4).

**Table 4:** LME's Similarity Percentage (SIMPER) explaining taxa contributing most to differences among LME.

	GoA	AgC	CBS	NCS	CbS	BgC	MdS	StS	SCS	PaS	KuC	GoC
**GoA**		Patellidae Corallinaceae Siphonariidae	Littorinidae Fucaceae Trochidae	Fucaceae Littorinidae Ulvaceae	Rhodomelaceae Corallinaceae Dictyotaceae	Patellidae Corallinaceae Mytilidae	Rhodomelaceae Patellidae Corallinaceae	Fucaceae	Chthalamidae Ostreidae Patellogastropoda	Rhodomelaceae Mytilidae Ulvaceae	Halichondriidae Patellogastropoda Polychaeta	Chthalamidae Neritidae Turridae
**AgC**	Phaeophyta Chlorophyta Rhodophyta		Littorinidae Fucaceae Trochidae	Fucaceae Littorinidae Cladophoraceae	Dictyotaceae Echinometridae Zoanthidae	Patellidae Sabellariidae Cryptonemiaceae	Dictyotaceae Cystoseiraceae Chlorophyta	Fucaceae	Ostreidae Patellogastropoda Chthalamidae	Chlorophyta Hildenbrandiaceae Balanidae	Patellogastropoda Lottiidae Polychaeta	Neritidae
**CBS**	Phaeophyta Chlorophyta Rhodophyta	Patellidae Corallinaceae Siphonariidae		Ulvaceae Mytilidae Lottiidae	Corallinaceae Dictyotaceae Echinometridae	Patellidae Mytilidae Buccinidae	Dictyotaceae Cystoseiraceae Mytilidae	Fucaceae	Ostreidae Chthalamidae Patellogastropoda	Mytilidae Chlorophyta Ulvaceae	Halichondriidae	Neritidae
**NCS**	Phaeophyta Chlorophyta Rhodophyta	Patellidae Corallinaceae Siphonariidae	Trochidae Patellidae Littorinidae		Corallinaceae Dictyotaceae Echinometridae	Patellidae Corallinaceae Trochidae	Patellidae Corallinaceae Dictyotaceae	Fucaceae	Chthalamidae Ostreidae Patellogastropoda	Chlorophyta Balanidae Siphonariidae	Halichondriidae Patellogastropoda Polychaeta	Chthalamidae Neritidae
**CbS**	Phaeophyta Chlorophyta Rhodophyta	Patellidae Corallinaceae Siphonariidae	Fucaceae Littorinidae Trochidae	Fucaceae Littorinidae Ulvaceae		Patellidae Mytilidae Trochidae	Patellidae Cystoseiraceae Chthalamidae	Fucaceae	Ostreidae Chthalamidae Patellogastropoda	Mytilidae Ulvaceae Chlorophyta	Halichondriidae Patellogastropoda Lottiidae	Chthalamidae Neritidae Turridae
**BgC**	Phaeophyta Chlorophyta Rhodophyta	Corallinaceae Siphonariidae Rhodomelaceae	Littorinidae Fucaceae Rhodomelaceae	Fucaceae Littorinidae Ulvaceae	Rhodomelaceae Dictyotaceae Echinometridae		Rhodomelaceae Dictyotaceae Cystoseiraceae	Fucaceae	Ostreidae Chthalamidae Patellogastropoda	Rhodomelaceae Chlorophyta Balanidae	Halichondriidae Patellogastropoda Lottiidae	Chthalamidae Neritidae Turridae
**MdS**	Phaeophyta Rhodophyta Chlorophyta	Patellidae Corallinaceae Siphonariidae	Fucaceae Littorinidae Trochidae	Fucaceae Littorinidae Ulvaceae	Corallinaceae Echinometridae Ralfsiaceae	Patellidae Buccinidae Actiniidae		Fucaceae	Ostreidae Patellogastropoda Chthalamidae	Ulvaceae Hildenbrandiaceae Siphonariidae	Halichondriidae Patellogastropoda Lottiidae	Neritidae
**StS**	Phaeophyta Chlorophyta Rhodophyta	Corallinaceae Patellidae Siphonariidae	Littorinidae Trochidae Rhodomelaceae	Littorinidae Ulvaceae Rhodomelaceae	Corallinaceae Rhodomelaceae Dictyotaceae	Patellidae Corallinaceae Mytilidae	Corallinaceae Patellidae Rhodomelaceae		Chthalamidae Ostreidae Patellogastropoda	Mytilidae Rhodomelaceae Ulvaceae	Halichondriidae Patellogastropoda Lottiidae	Chthalamidae Neritidae
**SCS**	Phaeophyta Chlorophyta Rhodophyta	Corallinaceae Patellidae Siphonariidae	Fucaceae Trochidae Littorinidae	Fucaceae Ulvaceae Littorinidae	Corallinaceae Rhodomelaceae Dictyotaceae	Patellidae Corallinaceae Mytilidae	Corallinaceae Patellidae Rhodomelaceae	Fucaceae		Mytilidae Rhodomelaceae Ulvaceae	Halichondriidae	Neritidae
**PaS**	Phaeophyta Rhodophyta Sessilia	Patellidae Corallinaceae Trochidae	Littorinidae Fucaceae Trochidae	Fucaceae Littorinidae Lottiidae	Corallinaceae Dictyotaceae Echinometridae	Patellidae Trochidae Buccinidae	Patellidae Dictyotaceae Cystoseiraceae	Fucaceae	Ostreidae Patellogastropoda		Halichondriidae	Neritidae
**KuC**	Phaeophyta Chlorophyta Rhodophyta	Corallinaceae Patellidae Siphonariidae	Littorinidae Fucaceae Trochidae	Fucaceae Littorinidae Ulvaceae	Corallinaceae Rhodomelaceae Dictyotaceae	Patellidae Mytilidae Corallinaceae	Corallinaceae Patellidae Rhodomelaceae	Fucaceae	Chthalamidae Ostreidae Patellogastropoda	Mytilidae Rhodomelaceae Ulvaceae		Chthalamidae Neritidae Turridae
**GoC**	Phaeophyta Chlorophyta Rhodophyta	Corallinaceae Patellidae Siphonariidae	Littorinidae Fucaceae Trochidae	Fucaceae Littorinidae Ulvaceae	Corallinaceae Rhodomelaceae Dictyotaceae	Patellidae Mytilidae Corallinaceae	Corallinaceae Patellidae Rhodomelaceae	Fucaceae Corallinaceae	Ostreidae Patellogastropoda Chthalamidae	Mytilidae Rhodomelaceae Ulvaceae	Halichondriidae Patellogastropoda Lottiidae	

**GoA**: Gulf of Alaska; **AgC**: Agulhas Current; **CBS**: Celtic-Biscay Shelf; **NCS**: Northeast U.S. Continental Shelf; **CbS**: Caribbean Sea; **BgC**: Benguela Current; **MdS**: Mediterranean Sea; **StS**: Scotian Shelf; **SCS**: Sotuh Chine Sea; **PaS**: Patagonian Shelf; **KuC**: Kuroshio Current; **SBS**: South Brazil Shelf and **GoF**: Gulf of California.

Comparisons are columns vs. rows, meaning families or taxa are more abundant in an LME by column compared to the LME of the intersecting row.

**Figure 4:**
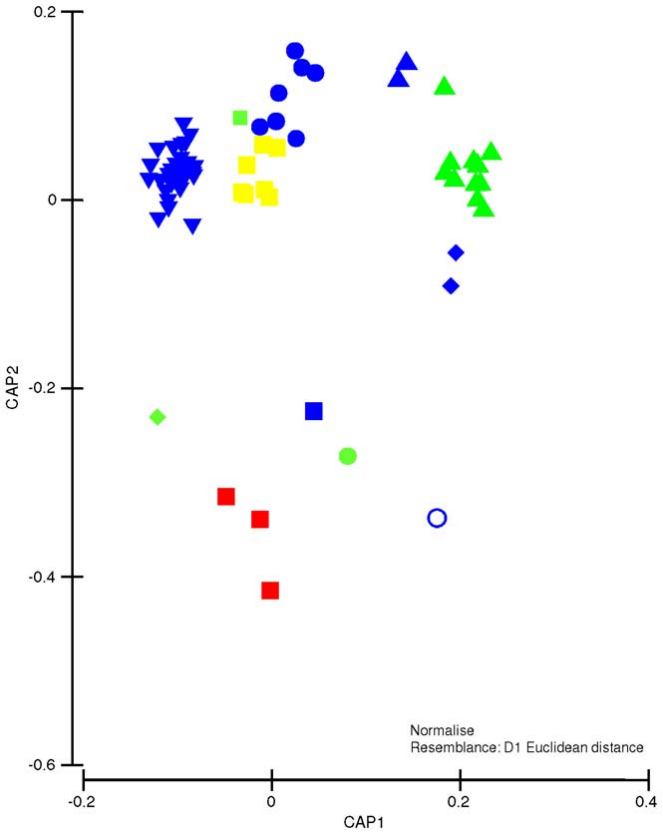
CAP on environmental data. Canonical analysis of principal coordinates (CAP) generated from Euclidian distances of the environmental matrix using LMEs as predictor factors. Green triangle  =  Gulf of Alaska, Yellow square  =  Agulhas Current, Red square  =  Mediterranean Sea, Blue triangle  =  Celtic-Biscay Shelf, Green diamond  =  Gulf of California, Blue diamond  =  Northeast US Continental Shelf, Inverted blue triangle  =  Caribbean Sea, Blue circle  =  Benguela Current, Green square  =  South China Sea, Green circle  =  Kuroshio Current, Blue square  =  Patagonian Shelf, Empty blue circle  =  Scotian Shelf.

A constrained ordination (CAP) of sites based on the environmental variables showed clear differences among sites located in different LMEs (Fig. 4). In the environmental ordination (Fig. 4), an important split over the second axis (δ_2_ = 99.5%) was not well related to any particular variable. Scores of the first axis (δ_1_ = 99.0%) were strongly and negatively correlated with SST (ρ = 0.68), indicating that sampling sites were ordered from left to right with decreasing SST (Fig. 4).

Correlation of the matrices of environmental variables with the biological, by means of a BIOENV routine, indicated that the variables that best explained patterns of spatial distribution of LMEs, based on their biological information (ρ = 61.1%) were: photoperiod, rain fall anomalies, SST, chlorophyll-a anomalies and the index of inorganic pollution (Table 5).

**Table 5:** Bio-ENV results showing the environmental variable combinations that best match the biotic similarity matrices using the weighted Spearman rank correlation (*ρ*_w_).

Number of variables considered	Correlation	Selections
5	0.611	PHO, RAla, SST, CHAa, INP
5	0.598	PHO, RAla, SST, CHAa, CHA
5	0.578	PHO, RAla, SST, CHA, INP
4	0.567	RAIa, SST, CHA, INP

## Discussion

The overall intertidal rocky shore assemblage descriptions provided here correspond well with documented species lists for some LME's (e.g. Caribbean Sea, Gulf of Alaska, and the South African Agulhas and Benguela Current), despite the often small sampling effort in our study. For the Caribbean, for example, similar assemblage description, based on dominant species, was obtained from more detailed studies with more effort [56]-[59]. This consistency is retained when spatial relationships were considered between the more southern Caribbean descriptions from this study (Fig. 1) with an intensive study of the British Virgin Island [56] in the northern Caribbean. Similarly, the general descriptions for Gulf of Alaska and the South African Agulhas and Benguela Currents LME assemblages matched published records based on more comprehensive sampling [60]–[66]. For some LMEs (e.g., Mediterranean Sea), three sites still produced a general description similar to what has been reported, especially in the northern Mediterranean Sea [25], [67], [68]. Hence, despite the low replication number per site, overall regional patterns in intertidal community structure seemed to be reasonable well captured in our study. While we emphasize that our available data were limited, they still seem to provide a useful database for this first-cut analysis of global patterns.

The proposed cline in species diversity from low to high latitudes for most terrestrial and some marine groups [69]–[72] is less consistent in the marine environment [73]–[75] or non-existent [31]. This study did not find a clear pattern in relation to latitude, especially in estimated species richness, a result that contrasts findings for algae [33] and intertidal echinoderms [76] from other NaGISA-based analyses. Macroalgae [33] and small intertidal echinoderms [76] had highest taxon richness in high northern latitudes. In contrast, large intertidal echinoderms diversity and abundance peaked in the Caribbean region [76]. It seems that different taxa may be structured differently along latitude. The complete assemblage may then not display any specific latitudinal trend as different gradients of the individual taxonomic components are averaged.

Alternatively, the lack of latitudinal patterns found in this study might be due to low sample size in some LMEs. Small sample sizes are likely to omit rare species in a given assemblage, which would result in underestimations of species richness for those particular LMEs.

In this study, despite the fact that no latitudinal gradient was found in terms of the univariate estimator of taxon richness, a clear latitudinal pattern was found for the multivariate aspect of taxonomic composition of intertidal rocky shores assemblages (Fig. 3). Similarity patterns among sampling sites were closely related to latitude but not with longitude. For example, sampling sites of the Kuroshio Current and South China Sea were grouped with sites in the Mediterranean Sea, which were all situated at similar latitudes (38°–40°N) yet on distinctly different longitudes. While it has been suggested before that in rocky shore environments, latitudinal patterns can be detected regionally while local patterns might be obscured by smaller-scale environmental variables or biological interactions [22], [77], the idea that latitudinal differences may be conserved across large longitudinal distances is novel and warrants further testing.

Differences in taxonomic composition among LMEs demonstrated that spatial distribution patterns of these assemblages were not homogeneous over large spatial scales. Consequently, null models, predicting uniform assemblage patterns over large spatial scales, could be discarded [78]. This leaves two alternative models: environmental models, where taxonomic composition is related to environmental variables (anthropogenic and/or natural, Table 2), and neutral models where taxonomic composition depends on geography (e.g., [37], [38]).

Through correlation analyses (i.e. BIOENV), six environmental variables were identified as potential drivers of spatial distribution patterns of intertidal rocky assemblages. Of those, five are considered “natural” variables, and only one (inorganic pollution index) was directly related to anthropogenic influences. There was no evidence to unequivocally separate environmental models and neutral models to explain taxonomic composition, because assemblages were highly correlated with latitude (Fig. 3; neutral model) as well as with SST and chlorophyll-a (environmental models). Noting that SST in this study was not strongly correlated with latitude (ρ = 0.38), it can be proposed that SST must play a key role in the observed global distribution patterns of these assemblages, as has been proposed on regional scales [16], [61], [75], [79]. The repercussions are of great importance since future changes in SST from climate change or global warming [17], [19] may alter the structure of these assemblages and, consequently, their functioning [80]. Another important environmental variable related to the patterns of spatial distribution of the natural assemblages was photoperiod, which might have a strong influence on the primary producers of these assemblages. Unfortunately, photoperiod is a function of latitude; consequently, an unequivocal separation between neutral and environmental models cannot be done. The direct effects of anthropogenic impacts such as pollution [81], food harvesting [82], eutrophication [83] and introduced species [84] on marine communities have been studied at regional and local scales. However, not many studies have attempted to associate intertidal rocky shore assemblage structure at a global scale with anthropogenic variables, although a global pervasive effects of human has been predicted for these environments (e.g., [83]). The lack of relationship of rocky intertidal assemblages with variables related to direct anthropogenic influences in this study might be due to the resiliency of some rocky shore organisms to contaminants such as high concentrations of heavy metals [85], [86] and oil spills [87]. Alternatively, the absence of significant correlations with variables related to direct anthropogenic influences could result from the level of accuracy and/or precision of the models used to estimate the different indexes (e.g., fisheries, invasive species, nutrients, etc.) since all variables were taken from one source [45]. For example, the model used to estimate impacts of the fisheries at a global scale has received criticism [88].

Correlative analysis does not establish cause and effect. However, the identification of correlated drivers can give us some insight into which variables may be most influential. Actual cause-consequences relationships between environmental (anthropogenic or natural) drivers and rocky shore assemblages at global scales are further complicated due to the inherent complexity of spatial and temporal variation in which these assemblages naturally fluctuate [89]. Furthermore, due to our current logistic limitations to do manipulative experiments at regional or global scales, the best and perhaps only way to understand the underlying processes that affect coastal bio-geographic distribution patterns is through large-scale and continuous monitoring programs. Therefore, it is imperative to continue global-scale programs to detect and characterize these changes over continued time series. Unfortunately, monitoring programs are traditionally seen as “Science's Cinderella” [90] and, consequently, do not receive the needed attention [91]. Despite the caveats of the data used in this study, we have shown the importance of generating global databases of biological information to gain a better understanding of the structure and functioning of rocky shore assemblages.

## Supporting Information

Table S1 (0.03 MB DOC)
