# Supplementary material for: Patterns of Spatial Variation of Assemblages Associated with Intertidal Rocky Shores: A Global Perspective
Source: PLoS One. 2010 Dec 16;5(12):e14354. doi: 10.1371/journal.pone.0014354 (PMC3002908; doi:10.1371/journal.pone.0014354)
Supplement: Table S1 — (0.03 MB DOC) [file pone.0014354.s001.doc]

Table S1. Permutational Multivariate Analyses of Variance (PERMANOVA) based on taxonomic dissimilarity indexes (Theta) calculated on presence/absence of species per quadrate (521 variables) including estimates of pseudo multivariate variation at each spatial scale and expressed as a proportion of the total. Analysis was done with 4999 permutations of residuals under a reduced model.

| **Source** | **d.f.** | **MS** | **Pseudo-F** | **P** | **C.V (%)** |
| --- | --- | --- | --- | --- | --- |
| LME | 8 | 31169 | 3.3392 | 0.0001 | 29,2 |
| Site(LME) | 21 | 9684.9 | 4.8607 | 0.0001 | 28,8 |
| Zone(Site(LM)) | 33 | 1861.8 | 6.1103 | 0.0001 | 19,0 |
| Res | 441 | 304.7 |  |  | 23,0 |
